# Evolving patterns of hepato-pancreato-biliary surgery in the immunotherapy era

**DOI:** 10.3389/fimmu.2026.1862622

**Published:** 2026-09-28

**Authors:** Ya’ara Gutman, Ofer Margalit, Shira Golan, Ron Pery, Rony Eshkenazy, Talia Golan, Ido Nachmany, Niv Pencovich

**Affiliations:** 1 Department of General Surgery and Transplantation, Sheba Medical Center, Tel-Hashomer, Gray Faculty of Medicine and Health Sciences, Tel-Aviv University, Tel-Aviv, Israel; 2 Department of Oncology, Sheba Medical Center, Tel-Hashomer, Gray Faculty of Medicine and Health Sciences, Tel-Aviv University, Tel-Aviv, Israel

**Keywords:** cancer, hepatopancreatobiliary surgery, immune modulation, immune system, immunotherapy, metastasis

## Abstract

**Introduction:**

Immunotherapy has revolutionized cancer treatment with remarkable outcomes, expanding treatment options and enabling new surgical opportunities for patients with previously unresectable disease. This descriptive study examines the impact of immunotherapy on the volume and nature of hepato-pancreato-biliary (HPB) surgeries in a large referral center and reviews the current literature.

**Methods:**

Analysis included consecutive surgeries performed in a referral HPB unit between January 1, 2020, and June 1, 2025. Patients undergoing HPB surgery following immunotherapy-based treatment, in clinical scenarios historically infrequently considered for surgery, were identified. Surgical volume trends, indications, perioperative outcomes, and oncologic follow-up were assessed.

**Results:**

Of 1,306 surgeries, 22 (1.6%) were performed in patients who received immunotherapy-based treatment prior to surgical consideration. The proportion of these patients increased from 0.71% in 2020 to over 4% in 2025. Surgical indications were divided into three main patterns; patients who were downstaged to resectability, those in whom the primary disease was controlled by immunotherapy and developed new resectable cancer, and those in whom surgery was used as an adjunct to immunotherapy. Notably, the proportion of complex procedures increased over the study period, reflecting growing surgical experience and confidence in the durability of immunotherapy responses.

**Discussion:**

Immunotherapy is reshaping the role of HPB surgery by expanding the spectrum of clinical scenarios in which surgical intervention may be considered. These findings highlight the evolving integration of surgery within multidisciplinary cancer care in the immunotherapy era and the need for refined patient selection and clearer operative indications.

## Introduction

Immunotherapy has expanded therapeutic options across malignancies by harnessing the immune system to target cancer cells. In the realm of hepato-pancreato-biliary (HPB) malignancies, traditional treatment options have often been limited, and patients presenting with initially unresectable tumors rarely reached surgery following systemic treatments. Immune checkpoint inhibitors (ICIs) targeting programmed cell death protein 1 (PD-1) and its ligand (PD-L1), alone or combined with anti-angiogenic agents or tyrosine kinase inhibitors, have introduced possibilities for conversion surgery in a subset of patients with previously non-operable HPB malignancies, particularly hepatocellular carcinoma (HCC), by inducing sufficient tumor regression to permit resection. In addition, emerging evidence also suggests a therapeutic role for immunotherapy in cholangiocarcinoma (CCA), alongside early and evolving evidence exploring its role in pancreatic ductal adenocarcinoma (PDAC) (1–6). Durable immunotherapy-mediated disease control may also allow second primary malignancies to become clinically apparent and surgically treated. Overall, the immunotherapy era is increasingly associated with new surgical scenarios for patients with oncological diseases that historically would not be considered for surgery.

In this study, we assess the impact of immunotherapy on the surgical practice of a referral HPB unit over a 5-year period. We evaluate evolving patterns of HPB surgery arising in the context of immunotherapy-based cancer care among patients who historically would rarely have been considered surgical candidates, and assess the outcomes of these surgeries. We also performed a literature review to assess whether the local pattern reflects a global trend.

## Materials and methods

### Study design and patient selection

This retrospective descriptive study included all patients who underwent elective or semi-elective HPB surgery at a tertiary referral center between January 1, 2020, and June 1, 2025. All oncological cases were reviewed during weekly multidisciplinary team (MDT) meetings comprising oncologists specializing in pancreas, biliary tract, and liver cancer, radiation oncologists, radiologists, HPB surgeons, upper gastrointestinal surgeons, and gastroenterologists.

Patients were eligible for inclusion in the study cohort if they underwent HPB surgery in a clinical context associated with immunotherapy-based cancer management.

### Data collection

Data were retrieved retrospectively using MDClone’s ADAMS platform, a self-service query tool that provides comprehensive patient-level data of wide-ranging variables in a defined time frame around an index event (http://www.mdclone.com). Collected data included patient demographics, medical history, comorbidities, tumor characteristics on imaging, details of immunotherapy and other oncological treatments, perioperative laboratory indices, surgical pathology, postoperative complications, adjuvant treatments, disease recurrence, and survival outcomes. All extracted data were manually reviewed and validated. Non-coded information, including imaging data, MDT decisions, treatment- and surgery-related complications, and additional clinical details, was manually retrieved from patient medical charts. Postoperative complications were classified according to the Clavien-Dindo (CD) system, with major complications defined as CD grade > IIIa (7). The interval between initiation of immunotherapy-based treatment and surgery, as well as preoperative Eastern Cooperative Oncology Group (ECOG) performance status, were collected and reported descriptively. Free-text screening and preliminary classification of selected retrospective variables were assisted by large language models (ChatGPT, OpenAI; and Claude, Anthropic). Model-assisted outputs used for the analysis were reviewed, corrected, and clinically adjudicated by the authors, and did not replace manual clinical review. ChatGPT was also used to improve grammar and language clarity. All study procedures adhered to the principles of the Declaration of Helsinki. The institutional review board at Sheba Medical Center approved the study (approval reference number SMC-9498-22), and the ethics committee waived the requirement for informed consent due to the retrospective nature of the research.

### Historical and contemporary comparative cohorts

To provide clinical context for the observed surgical scenarios, we additionally identified diagnosis-based cohorts from two time periods: a historical pre-modern immunotherapy period (2010–2015) and the contemporary study period (2020–2025). This analysis was distinct from the primary surgical cohort, which was derived from all HPB surgical procedures performed during the contemporary study period.

The comparative analysis focused on the major malignancies represented in our cohort and most relevant to HPB surgical practice, including HCC, cholangiocarcinoma, gallbladder carcinoma, and pancreatic/periampullary malignancies. Melanoma was evaluated separately because it was represented in our cohort but it is not an HPB primary malignancy and may follow different patterns of dissemination, treatment response, and surgical management.

For each disease category, we assessed the total number of diagnosed patients, exposure to immunotherapy, documentation of metastatic disease, and subsequent HPB surgical intervention. Metastatic status was determined from oncology-derived structured M-staging fields when available, supplemented by text-based clinical and pathological documentation of distant metastatic disease. HPB surgical interventions included hepatic, biliary, pancreatic, and periampullary resections, biliary or gastric bypass procedures, diagnostic or exploratory HPB operations, and aborted HPB surgical exploration. For the metastatic surgical analysis, interventions were counted only when metastatic disease was documented before or at the time of surgery. Procedures with a resection component were identified separately and used as a surrogate for potentially curative or definitive surgical intent. These analyses were intended to provide clinical context and were not designed for formal causal inference.

To further characterize the contemporary treatment-to-surgery pathway, we performed an additional focused analysis among patients with documented metastatic HPB-primary malignancy or melanoma who received immunotherapy. Structured MDT records and free-text oncology notes were reviewed to identify MDT discussion after immunotherapy initiation; we then assessed how many patients subsequently underwent HPB surgery. Formal surgical candidacy, reasons for non-operative management, and progression before planned surgery were not treated as extractable variables because they were not consistently documented in structured fields.

### Statistical analysis

Given the exploratory nature of the study and the heterogeneity of the cohort, analyses were primarily descriptive.

## Results

Between January 1, 2020, and June 1, 2025, 1,306 patients underwent HPB surgery performed by four HPB surgeons. Seventy-five patients (5.7%) had received immunotherapy at some point during their oncologic course. After excluding patients in whom immunotherapy was administered for postoperative recurrence or adjuvant indications, 22 patients remained whose surgical indication was considered to be associated with immunotherapy-based disease management.

The relationship between immunotherapy and surgery varied substantially across patients and included both direct oncologic conversion to resectability and indirect surgical implications of durable disease control. To capture the heterogeneity of clinical scenarios, patients were categorized into three groups according to the mechanism by which immunotherapy facilitated surgical intervention:

Group I: surgery for initially unresectable disease downstaged by immunotherapy (n=13, 59%):

These patients initially presented with locally advanced or metastatic disease, which rendered them unresectable, and were therefore referred for immunotherapy. Following treatment, their disease demonstrated sufficient response or durable stabilization to permit surgical consideration (see representative case in **Figure 1A**).

**Figure 1:**
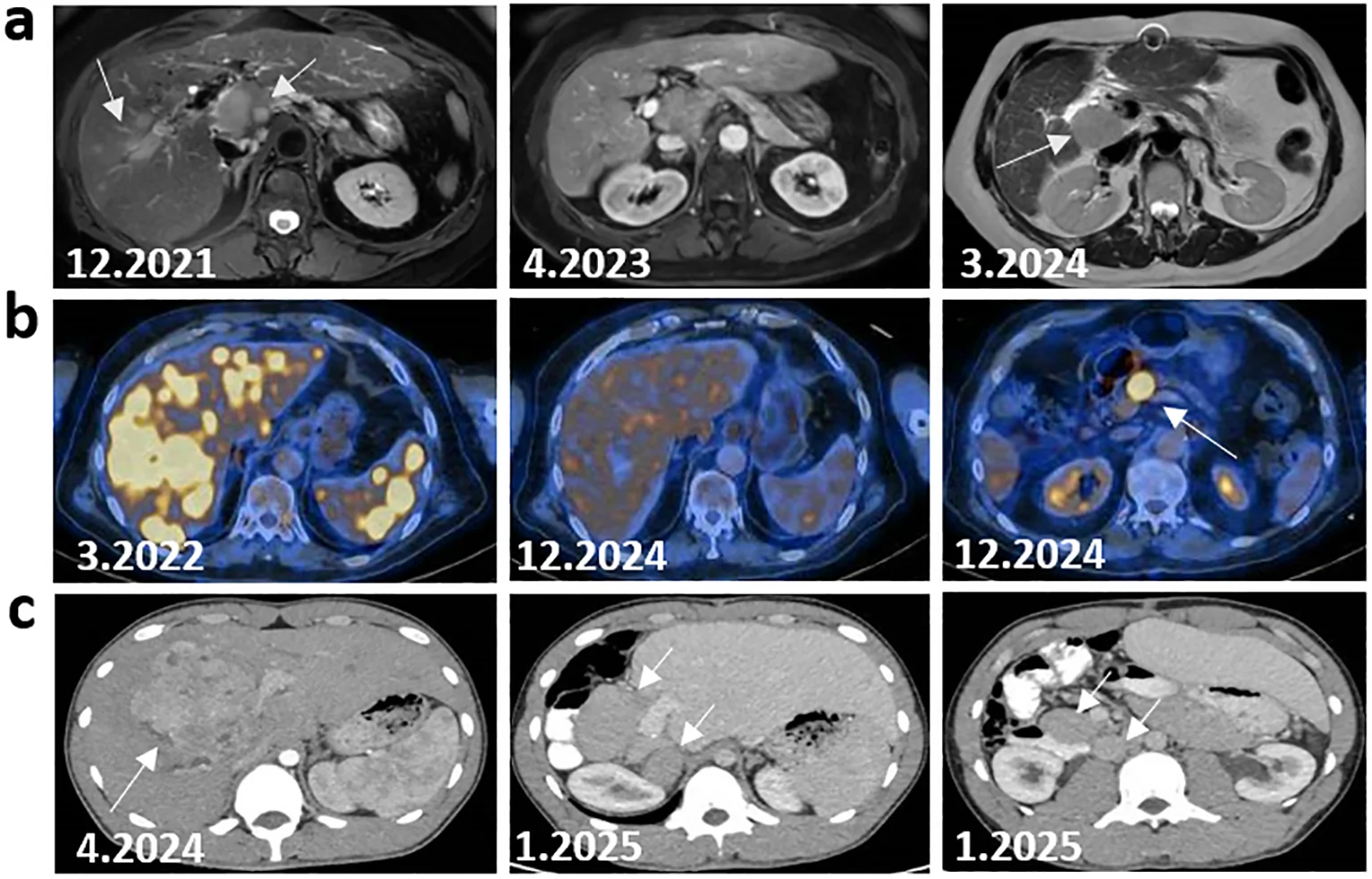
Patterns of surgical indications. Imaging of representative cases from the three patient groups, demonstrating the variability of diagnoses, surgical scenarios and indications for surgery. **(a)** Group I – downstaging: Serial MRI of a 70-year-old woman with multifocal bilobar hepatocellular carcinoma diagnosed in December 2021. After 14 months of atezolizumab–bevacizumab therapy, imaging demonstrated complete hepatic response. In March 2024, enlargement of a portocaval lymph node prompted lymphadenectomy; pathology revealed metastatic gastrointestinal/pancreatobiliary adenocarcinoma. The patient remains disease-free at 15 months. **(b)** Group II – second primary malignancy: A patient with widely metastatic melanoma who achieved durable response to immunotherapy (pembrolizumab followed by ipilimumab–nivolumab). CT and FDG-PET initially demonstrated multiple FDG-avid liver metastases that subsequently became metabolically inactive, while a pancreatic lesion appeared. Biopsy confirmed pancreatic ductal adenocarcinoma, and the patient underwent pancreaticoduodenectomy. The patient was disease-free at 10 months. **(c)** Group III – adjunct to immunotherapy: CT images of a 23-year-old man with fibrolamellar HCC. Initial imaging showed a large right hepatic mass with retroperitoneal lymph node involvement. Following extended right hepatectomy and lymphadenectomy, surveillance imaging was initially negative, but recurrence with pulmonary and intra-abdominal metastases was detected in January 2025. The patient was enrolled in a phase I trial evaluating a DNAJB1–PRKACA fusion–based peptide vaccine combined with immune checkpoint inhibition, with debulking surgery performed prior to treatment initiation (16). CT, computed tomography; FDG-PET, fluorodeoxyglucose-18 positron emission tomography; MRI, magnetic resonance imaging.

Group II: surgery for a second primary malignancy in patients with controlled advanced disease (n=2, 9%):

These patients were diagnosed with advanced oncologic disease and achieved durable systemic disease control under immunotherapy-based treatment. During this prolonged period of disease control, second primary malignancies became clinically apparent and subsequently underwent surgical resection (see representative case in **Figure 1B**).

Group III: surgery as an immunotherapy-adjunct in patients with controlled advanced disease (n=7, 31.8%):

Patients with controlled or partially controlled advanced disease who underwent surgery for complementary indications, such as debulking, management of sanctuary-site disease, or treatment of recurrent biliary complications (see representative case in **Figure 1C**). In this group, surgery was integrated into longitudinal multidisciplinary disease management rather than performed with classic curative intent alone. These procedures reflected evolving patterns of chronic oncologic care in the immunotherapy era.

To further illustrate the heterogeneity of clinical scenarios included in this cohort, **Supplementary Table 1** summarizes the individual patient-level clinical pathways, surgical interventions, and proposed mechanisms linking immunotherapy-based cancer management and surgery across all three groups.

The proportion of HPB surgeries performed in the context of immunotherapy-based cancer management increased markedly over the study period, from less than 0.7% of all cases in 2020 to more than 4% in 2025 (see **Figure 2**). Patient demographics, cancer characteristics, immunotherapy, and surgery data are depicted in **Table 1**. Sixteen patients (72%) were male, with a median age of 58 years (interquartile range (IQR) 52-71). Most patients had significant comorbidity (American Society of Anesthesiologists (ASA) score ≥III). Baseline diagnosis was variable, including HPB malignancies (HCC, CCA, gallbladder cancer and PDAC), as well as non-HPB malignancies such as melanoma, lung cancer and lymphoma. The median interval from initiation of immunotherapy-based treatment to surgery was 9 months (range, 0-72) among 21 evaluable patients. The interval was variable and tended to be longer in later study years. When grouped into early and late periods, the median interval increased from 3 months in 2020–2023 to 20 months in 2024-2025. Preoperative ECOG performance status was available for 14 patients; all had ECOG ≤2, including 11 patients with ECOG 0-1.

**Figure 2:**
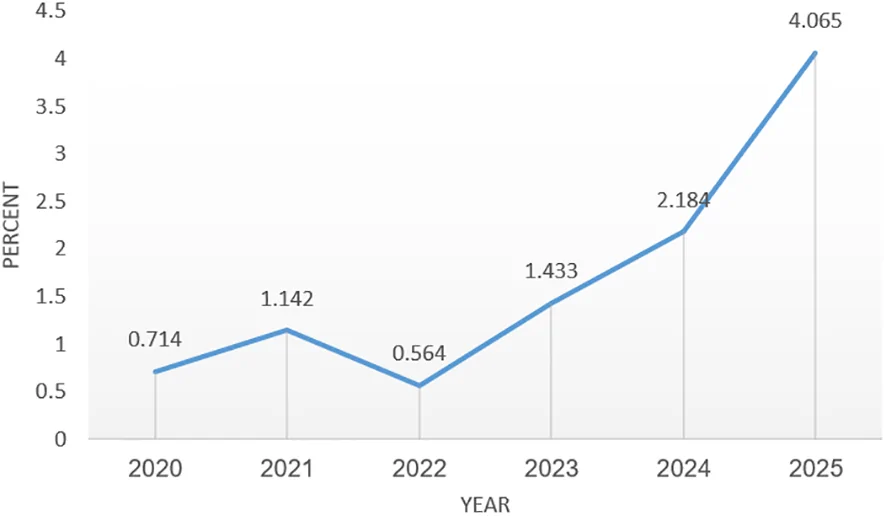
Trend in the proportion of HPB surgeries performed following immunotherapy from 2020 to 2025. Values represent the percentage of total annual HPB surgeries in one referral center, demonstrating a progressive increase over time.

**Table 1:** Patients’ demographic and surgical characteristics.

	Group I: downstaging, n=13	Group II: second primary, n=2	Group III: adjunctive, n=7
	Number (%) or median (range)	Number (%) or median (range)	Number (%) or median (range)
Age, years	58 (45-75)	75 (73-77)	56 (22-71)
Male sex	10 (76.9%)	1 (50%)	5 (71.4%)
BMI	24.2 (16.3-35.1)	26.1 (25.2-27)	23.6 (17.4-26.2)
ASA score, n ^A^			
- 2	1	0	0
- 3	10	2	6
- 4	0	0	1 ^B^ (ASA 4E)
Type of malignancy			
- Cholangiocarcinoma	3 (23%)	0	0
- Gallbladder cancer	1 (7.7%)	0	0
- Pancreatic ductal adenocarcinoma (PDAC)	2 (15.3%)	1 (50%)	0
- Hepatocellular carcinoma (HCC)	2 (15.3%)	0	2 (28.5%)
- Melanoma	2 (15.3%)	0	1 (14.3%)
- Renal cell carcinoma (RCC)	1 (7.7%)	0	2 (28.5%)
- Colorectal carcinoma (CRC)	2 (15.3%)	1 (50%)	0
- Other ^C^	0	0	2 (28.5%)
Disease extent before immunotherapy			
- Localized/resectable	0	2 (100%)	0
- Locally advanced or borderline resectable	4 (30.8%)	0	0
- Metastatic	9 (69.2%)	0	7 (100%)
Type of immunotherapy used, n ^D^			
- Nivolumab alone	2	1	0
- Ipilimumab + Nivolumab	2	1	3
- Pembrolizumab	2	1	1
- Atezolizumab	2	1	3
- Durvalumab	4	0	0
- Other (trial medications)	1	0	2
Type of surgery, n			
- Hepatectomy	1	1	2
- Adrenalectomy	2	0	1
- Lymphadenectomy	2	0	1
- HPD	2	0	0
- Pancreaticoduodenectomy	1	0	1
- Distal pancreatectomy with splenectomy	1	1	0
- Hepaticojejunostomy	0	0	1
- Splenectomy	0	0	1
- Radical nephrectomy with IVC thrombectomy	1	0	0
- Aborted procedure	3	0	0
Surgery intent			
- Palliative	1 (7.7%)	0	5 (71.4%)
- Curative	12 (92.3%)	2 (100%)	2 (28.5%)
Resection margins (R status)			
- R0	8 (61.5%)	2 (100%) ^E^	2 (28.5%)
- R1	2 (15.3%)	0	0
- R2	0	0	3 (42.8%)
- No resection performed ^F^	3 (23%)	0	2 (28.5%)
Intraoperative complications	2 (15.3%)	0	2 (28.5%)
Postoperative complications			
- Major (Clavien-Dindo > IIIa)	2 (15.3%)	0	0
- Minor (Clavien-Dindo ≤ IIIa)	7 (53.8%)	0	2 (28.5%)

ASA, American Society of Anesthesiologists; BMI, body mass index; HPD, hepatopancreatoduodenectomy; IVC, inferior vena cava.

^A^ASA score was unavailable for 2 patients; counts are shown without percentages.

^B^This patient underwent emergent palliative formal right hepatectomy due to sepsis that developed after angioembolization of massive hemorrhage from hepatocellular carcinoma.

^C^Other: small-cell lung cancer (n=1), diffuse large B-cell lymphoma (n=1).

^D^Several patients received more than one immunotherapy regimen, either in combination or sequentially; counts are shown without percentages.

^E^One patient had a pathological complete response (pCR).

^F^No resection was performed in five cases: three aborted procedures, one palliative hepaticojejunostomy, and one liver biopsy for tumor-infiltrating lymphocyte (TIL) therapy.

Before immunotherapy initiation, 16 patients (72.7%) had metastatic disease and 4 (18.2%) had locally advanced or borderline resectable tumors. Two patients had resectable disease, both from Group II, the second-primary subgroup (see **Supplementary Table 1**).

Immunotherapy regimens varied according to primary diagnosis (**Table 1**).

Mismatch repair deficiency or high microsatellite instability was present in 23% of Group I patients (n=3). Pathological response to immunotherapy-based treatment was not systematically reported across the cohort. Documented pathological complete response was identified in two patients (G1-2 and G1-4, **Supplementary Table 1**): one patient with renal cell carcinoma (RCC) treated with ipilimumab-nivolumab prior to radical nephrectomy, and one patient with microsatellite instability-high (MSI-H)/Lynch-associated pancreatic ductal adenocarcinoma (PDAC) treated with neoadjuvant chemo-immunotherapy prior to pancreaticoduodenectomy. In several additional cases, extensive tumor necrosis was described; however, in the absence of standardized pathological response assessment, these findings could not be reliably attributed to immunotherapy.

### Surgical procedures and perioperative outcomes

The most common procedures were liver resection (n=5), pancreatectomy (n=4), and hepatopancreatoduodenectomy (HPD; n=2). Notably, no hepatectomies were performed in this cohort before 2023. The frequency of hepatectomy increased thereafter, paralleling growing institutional experience, broader adoption of immunotherapy, and increased referral of patients for surgical consideration. Other procedures included lymphadenectomy (n=3) and adrenalectomy (n=3), among others (**Table 1**). Sixteen procedures were performed with curative intent, of which ten achieved complete resection. Three operations (13.6%) were aborted due to unexpected intraoperative findings (vascular involvement in a patient with PDAC, hepatic dissemination in a patient with CCA, and extensive nodal spread with involvement of the right anterior portal pedicle in a patient with colorectal liver metastasis (CRLM)). Intraoperative complications occurred in four patients, including hemorrhage in three and dehiscence of a previous biliary anastomosis in one. Postoperative complications were recorded in 11 patients (50%), including major complications (CD > IIIa) in two patients (9.1%).

At last follow-up, with a median follow-up of 16.5 months (range 1-61), 7 patients were alive without evidence of disease, 9 were alive with disease, 4 had died of disease progression, and 2 had died of other causes. Given the small size and heterogeneity of the cohort, formal comparison of oncologic outcomes between the different groups was not performed.

### Historical and contemporary diagnosis-based comparison

To place these cases in historical context, we compared diagnosis-based cohorts from 2010–2015 and 2020-2025, focusing on HPB-primary malignancies represented in our cohort; melanoma was analyzed separately. In HPB primaries, immunotherapy exposure increased from 5/714 patients (0.7%) to 122/1,312 (9.3%); among metastatic patients, it increased from 1/186 (0.5%) to 71/568 (12.5%).

HPB interventions increased from 100/714 patients (14.0%) to 265/1,312 (20.2%) overall, and from 14/186 (7.5%) to 64/568 (11.3%) among metastatic patients. In contrast, HPB interventions among metastatic melanoma patients decreased from 4/82 (4.9%) to 7/377 (1.9%). Among metastatic HPB-primary patients undergoing HPB intervention, resectional procedures, used as a surrogate for potentially curative or definitive intent, increased from 3/14 (21.4%) to 27/64 (42.2%).

To further characterize the contemporary treatment-to-surgery pathway, we performed an additional focused analysis of metastatic patients with HPB-primary malignancy or melanoma who received immunotherapy during the contemporary study period. This analysis identified 396 patients, of whom 256 (64.6%) had documented MDT discussion after immunotherapy initiation. Overall, 24 patients (6.1%) proceeded to HPB surgery. Formal surgical candidacy without subsequent operation and progression before planned surgery could not be reliably extracted, because these events were not consistently documented as structured variables or clearly linked to specific surgical decision points in the free-text records. These findings provide a partial description of the treatment-to-surgery pathway but should not be interpreted as a complete surgical-candidacy funnel.

## Discussion

In the last two decades, immunotherapy has evolved dramatically and revolutionized the management and outcomes of many malignancies (8). In the present study, we describe an emerging interface between immunotherapy and the landscape of HPB surgery. Over a relatively short time frame, immunotherapy expanded the spectrum of patients and clinical scenarios in which HPB surgery could be considered. At our institution, “immunotherapy-associated procedures” increased from less than 1% of all HPB resections in 2020 to more than 4% by 2025, accompanied by a gradual expansion in operative scope, including major hepatectomies and complex combined resections. This temporal increase should be interpreted as part of a broader change in multidisciplinary oncologic practice, rather than as a direct treatment effect of immunotherapy alone. Wider immunotherapy use, evolving referral patterns, accumulated surgical experience, and increasing confidence in durable disease control likely all contributed to the growing consideration of HPB surgery in selected patients. Perioperative morbidity remained within expected ranges and the rate of aborted procedures was low, despite the advanced oncologic profile of this cohort. These observations suggest that integration of immunotherapy into surgical pathways is feasible in selected patients and reflects an evolving role for surgery within multidisciplinary oncologic practice.

Importantly, the interaction between immunotherapy and surgery in this cohort was heterogeneous and should not be interpreted as a single biologic entity. Accordingly, this study should be interpreted primarily as a descriptive analysis of evolving surgical patterns in the immunotherapy era.

While more than half of the patients underwent surgery following favorable response to immunotherapy-based treatment, additional scenarios emerged. Notably, whereas most existing reports focus primarily on downstaging and neoadjuvant paradigms, our cohort captures a broader spectrum of clinical indications for surgery in the immunotherapy era. Durable systemic disease control and prolonged survival may also allow the emergence or clinical detection of second primary malignancies that previously would not have been encountered during the patient’s disease course, thereby enabling curative resection of second primary malignancies in selected patients, a scenario rarely encountered prior to the immunotherapy era due to limited survival. In other cases, surgery was performed as an adjunct to ongoing immunotherapy for progressive disease, persistent nodal involvement, adrenal metastases, or management of tumor-related complications. In this setting, most procedures were undertaken with non-curative, palliative intent, reflecting a distinct therapeutic role compared to conventional resection strategies.

The temporal cohort comparison provides additional context for interpreting these findings. Among patients with metastatic HPB primary malignancies, HPB surgical interventions increased modestly between the historical and contemporary periods, from 7.5% to 11.3%. A more notable difference was observed when focusing on the type of operation performed: procedures with a resectional component, used as a surrogate for potentially curative or definitive surgical intent, accounted for 21.4% of metastatic HPB interventions in the historical cohort compared with 42.2% in the contemporary cohort. In contrast, melanoma showed the opposite pattern, likely reflecting its distinct biology, patterns of dissemination, treatment response, and relationship to HPB surgical decision-making.

These findings are consistent with an evolving role for HPB surgery in selected patients during the immunotherapy era, but they do not establish causality. This comparison cannot determine whether individual patients would have become surgical candidates without immunotherapy, and it cannot separate the contribution of immunotherapy from changes in referral patterns, surgical experience, imaging surveillance, multidisciplinary decision-making, or other systemic therapy protocols. Accordingly, in the present study, the relationship between immunotherapy exposure and surgical indication was assessed by detailed patient-level review rather than inferred from cohort-level comparisons alone.

The additional treatment-to-surgery pathway analysis provides partial quantitative context for the selection process leading to HPB surgery. Among 396 metastatic immunotherapy-treated patients with HPB-primary malignancy or melanoma, 256 had documented MDT discussion after immunotherapy initiation, whereas only 24 proceeded to HPB surgery. These findings reinforce that patients ultimately undergoing HPB surgery represent a highly selected subgroup of immunotherapy-treated patients. At the same time, the identification of metastatic immunotherapy-treated patients proceeding to HPB surgery suggests that this population represents a clinically meaningful component of the evolving metastatic HPB surgical landscape. However, this observation cannot establish causality or determine whether immunotherapy itself enabled surgical intervention in individual patients. The analysis also clarified which elements could not be reliably retrieved from retrospective data. Formal surgical candidacy, reasons for non-operative management, and progression specifically before planned surgery were not consistently documented as structured variables or clearly linked to surgical decision points in free-text records. Accordingly, these data provide a partial treatment-to-surgery pathway rather than a complete prospective surgical-candidacy funnel. Future studies should include systematic MDT documentation of surgical consideration, reasons for non-operative management, progression before surgery, and final treatment decisions.

Historically, resectability in HPB oncology has been defined almost exclusively by static anatomic criteria, including vascular involvement, nodal spread and metastasis at diagnosis. Patients presenting with locally-advanced or metastatic tumors were rarely considered for surgery, even when partial responses were achieved. Increasingly, however, immunotherapy is contributing to a more dynamic assessment of biologic behavior over time. Similar to the patterns observed in our cohort, the literature increasingly describes cases in which immunotherapy-based systemic therapy results in downstaging of HPB malignancies, converting initially unresectable disease to resectable and enabling potentially curative surgery. In parallel, neoadjuvant immunotherapy strategies are being explored in patients with technically resectable disease, with emerging evidence suggesting that preoperative immune activation may enhance pathological response and improve oncologic outcomes (9, 10).

In HCC, multiple neoadjuvant and conversion immunotherapy studies have demonstrated that ICIs, alone or in combination with anti-angiogenic agents or locoregional therapies, can induce substantial pathological tumor regression (11). Major pathological response (MPR) and pathological complete response (pCR) have been reported even in patients whose radiographic responses appear modest. Pooled analyses report MPR and pCR rates of 23% and 19%, respectively, in patients with resectable HCC treated with neoadjuvant immunotherapy (9). Another study demonstrated a conversion rate of 15.9%, with 60% of these patients achieving a pCR, among initially unresectable HCC cases downstaged by immunotherapy (1). In cohort-level studies, pathological response and immune infiltration patterns have been associated with improved recurrence-free survival (2, 9, 11, 12). Early data in intrahepatic CCA similarly describe conversion of initially unresectable tumors to resectable disease in selected patients (13), and encouraging results have also been reported in studies of PDAC (4). Although pathological response, CD8+ tumor-infiltrating lymphocytes, and tertiary lymphoid structures have been associated with immunotherapy response and recurrence-free survival in selected neoadjuvant immunotherapy studies, these pathological and immune-response features were not systematically assessed in our cohort and therefore were not incorporated into the present analysis. This highlights the importance of standardized post-immunotherapy pathological documentation, including assessment of MPR and pCR, viable tumor percentage, immune-cell infiltration, and tertiary lymphoid structures, in future studies (10).

Together, these clinical and pathological observations introduce a clinically relevant concept of biologic selection. The treatment period functions as a “test of time” and “test of biology”, allowing tumors with sustained immune-mediated control to declare themselves. In HPB surgery, where operative morbidity can be considerable and the consequences of futile intervention may be significant, this strategy offers a means of expanding surgical candidacy.

As a concept, there is a strong biologic rationale for using immunotherapy in the neoadjuvant setting. Administration of ICIs while the primary tumor and its draining lymphatic basin remain intact may enhance antigen presentation and promote expansion of tumor-specific T-cell clones, generating systemic immune memory (14). This mechanism may be particularly relevant in HPB malignancies, where occult micrometastatic disease and early recurrence remain dominant failure patterns. The encouraging clinical outcomes observed in a subset of downstaged patients in our cohort are consistent with this biologic rationale, although prospective validation is required.

A recurrent challenge in the literature is the discordance between radiographic and pathological response following immunotherapy. Standard Response Evaluation Criteria for Solid Tumors (RECIST) criteria often fail to capture treatment effect, as immune infiltration, fibrosis, and edema may mimic pseudoprogression. Increasingly, MPR and pCR are being recognized as more reliable surrogates for long-term outcomes. From a surgical perspective, this repositions pathology as a central translational interface, providing both local control and high-resolution biological insight to guide prognosis and further therapy (12, 15).

In our cohort, a relatively high proportion of lymphadenectomies and adrenalectomies suggests that residual disease may persist in nodal basins or anatomical “sanctuary sites,” despite otherwise effective systemic control. Such patterns may reflect heterogeneity in immune penetration or local tumor microenvironment. In this context, surgery serves as a targeted strategy for eradication of immune-resistant niches.

The clinical role of surgery differed substantially across the various scenarios represented in this cohort. In a subset of patients, surgery was performed with curative intent following favorable response or downstaging under immunotherapy-based treatment. In other cases, surgery was performed with non-curative intent, for example to control disease-related complications such as recurrent cholangitis, which may historically have been encountered less frequently due to limited survival in advanced disease. These differences emphasize that surgery in the immunotherapy era should not be viewed as a uniform or solely curative strategy, but rather a spectrum of interventions with distinct biologic rationales and therapeutic goals.

The interval between initiation of immunotherapy-based treatment and surgery was variable and tended to be longer in later study years. This may reflect greater clinical familiarity with immunotherapy and longer survival under disease control. In addition, available preoperative ECOG data showed that patients undergoing surgery generally had preserved performance status, further supporting that this cohort represents a selected subgroup of patients. These observations reinforce the potential for guarantee-time bias. Patients necessarily had to survive long enough, maintain adequate clinical status, and remain suitable for operative consideration after immunotherapy-based treatment. Accordingly, the cohort represents a selected subgroup, and favorable outcomes should not be interpreted as evidence of survival benefit. Rather, these findings emphasize the importance of careful patient selection and the need for larger prospective studies to define which patients may benefit from surgical intervention in this setting.

This study is limited by its retrospective design, small sample size, and single-center nature. Given the small overall sample size and very small subgroup sizes, particularly Group II, the findings should be interpreted as exploratory and hypothesis-generating rather than inferential. In addition, the cohort included multiple malignancies with distinct tumor biology, immunotherapy responsiveness, and surgical indications. Accordingly, the study was not designed to evaluate disease-specific immunotherapy efficacy or compare oncologic outcomes across tumor types. The retrospective design and absence of fully matched comparator cohorts also limit the ability to establish definitive causality between immunotherapy and surgical candidacy. Standardized pathological response metrics, including MPR and pCR, were not systematically available. Nonetheless, this study provides real-world evidence that the expanding integration of immunotherapy and HPB surgery is feasible, biologically plausible, and oncologically meaningful in selected patients. In addition, the three-category framework proposed in this study - conversion to resectability, resection of second primary malignancies during durable disease control, and adjunctive surgery in controlled advanced disease - may provide a practical structure for future prospective studies evaluating surgical indications, timing of operative consideration, and patient selection after immunotherapy. As immunotherapy indications continue to expand, careful patient selection becomes increasingly critical to optimize outcomes while avoiding futile intervention. Future efforts should focus on defining appropriate clinical indications for surgery in this context and validating predictive biomarkers. Larger prospective studies with comparator cohorts will also be required to better characterize oncologic outcomes and clarify the evolving role of surgery in the immunotherapy era.

In conclusion, the landscape of HPB surgery is evolving in the immunotherapy era, with expanding clinical scenarios in which surgical intervention may be considered in selected patients. In our cohort, this evolution was reflected in a measurable increase in operative interventions, expansion of procedural complexity, and acceptable perioperative outcomes in patients historically deemed inoperable. Successful integration of immunotherapy and surgery will depend on sustained multidisciplinary collaboration and longitudinal assessment of tumor biology and treatment response. Future studies should focus on identifying the clinical and biological features that best define patients who may benefit from surgical intervention within immunotherapy-based cancer care.

## Data Availability

The data analyzed in this study is subject to the following licenses/restrictions: Unidentified patient data set used in this study is available from the corresponding author upon request. Requests to access these datasets should be directed to niv.pencovich@sheba.health.gov.il.
