# Supplementary material for: Evolving patterns of hepato-pancreato-biliary surgery in the immunotherapy era
Source: Front Immunol. 2026 Sep 28;17:1862622. doi: 10.3389/fimmu.2026.1862622 (PMC13656856; doi:10.3389/fimmu.2026.1862622)
Supplement: Supplementary file 1 [file Table1.pdf]

Supplementary Table 1: Clinical pathways linking immunotherapy-based cancer management and HPB surgery

**Group 1** - Direct conversion to resectability after immunotherapy				
Patient	Malignancy	Immunotherapy-associated disease course	Surgery performed	Proposed mechanism
G1-1	Multifocal HCC	Multifocal HCC treated with atezolizumab-bevacizumab with complete liver response and enlarging porto-caval node	Porto-caval lymphadenectomy	Resection of isolated residual/sanctuary-site disease
G1-2	HCC	Advanced HCC with recurrence after hepatectomy, responsive to Atezolizumab-Bevacizumab except adrenal	Left adrenalectomy	Sanctuary-site residual resectable disease
G1-3	Distal CCA	Initially locally advanced tumor treated with cisplatin-gemcitabine-durvalumab with favorable response	HPD (central hepatectomy)	Locally advanced CCA at presentation, with favorable response to immunotherapy
G1-4	Hilar CCA	CCA with suspected liver metastases, treated with cisplatin-	Right hepatectomy with bile duct resection	Conversion surgery after favorable systemic response

		gemcitabine-durvalumab		
G1-5	Intrahepatic CCA	Multifocal intrahepatic CCA treated with cisplatin-gemcitabine-durvalumab with radiographic and biochemical response	Diagnostic laparoscopy (aborted hepatectomy)	Attempted conversion surgery after systemic disease control
G1-6	Gallbladder carcinoma	Initially unresectable disease with peritoneal metastasis treated with cisplatin-gemcitabine-durvalumab with radiographic response	HPD (extended right hepatectomy) & right hemicolectomy	Conversion surgery after favorable response to immunotherapy with stable disease
G1-7	MSI-H PDAC (Lynch syndrome)	Resectable PDAC treated with neoadjuvant FOLFOX-pembrolizumab with complete pathological response	PD	Neoadjuvant immunotherapy-associated pathologic response
G1-8	MSI-H PDAC (Lynch syndrome)	Borderline resectable PDAC treated with neoadjuvant FOLFIRINOX-pembrolizumab with marked	Exploratory surgery (aborted PD)	Neoadjuvant immunotherapy-based management in biologically selected PDAC

		clinical and metabolic response		
G1-9	Metastatic CRC	Advanced metastatic CRC treated with investigational intratumoral immunotherapy with subsequent long-term disease control	Right adrenalectomy	Delayed sanctuary-site resection during prolonged survival after immunotherapy
G1-10	Recurrent CRC with CRLM	Longstanding recurrent CRC, this time CRLM, treated with nivolumab with durable metabolic response	Exploratory surgery (aborted hepatectomy)	Attempted resection after prolonged immunotherapy-mediated disease stabilization
G1-11	Metastatic melanoma	Durable complete response under checkpoint inhibition with isolated enlarging nodal recurrence	Common hepatic artery nodal resection	Sanctuary-site progression despite otherwise durable systemic control
G1-12	Metastatic melanoma	Prior durable response to pembrolizumab with isolated splenic hilar recurrence	Distal pancreatectomy and splenectomy	Localized progression during prolonged immunotherapy-mediated disease control
G1-13	Metastatic RCC	Advanced RCC treated with ipilimumab-nivolumab with major clinical	Radical nephrectomy with IVC reconstruction and colectomy	Conversion to resectability following durable systemic disease control

		and radiographic response		
**Group 2** - Second primary malignancies emerging during durable immunotherapy-mediated disease control				
Patient	Malignancy	Immunotherapy-associated disease course	Surgery performed	Proposed mechanism
G2-1	PDAC	Durable systemic control of metastatic melanoma under checkpoint inhibitor followed by development of resectable PDAC	Distal pancreatectomy and splenectomy	Second primary malignancy becoming clinically apparent during prolonged survival
G2-2	CRC with CRLM	Durable control of metastatic SCLC under chemo-immunotherapy followed by development of colon cancer with liver metastases	Non-anatomic hepatectomy	Prolonged survival allowing clinically significant second primary malignancy
**Group 3** - Surgery integrated into chronic immunotherapy-based oncologic management				
Patient	Malignancy	Immunotherapy-associated disease course	Surgery performed	Proposed mechanism
G3-1	Metastatic HCC	Hemorrhagic shock shortly after initiation of atezolizumab-bevacizumab	Right hepatectomy	Emergent palliative surgery during immunotherapy-based management
G3-2	Fibrolamellar HCC	Progressive metastatic disease	Debulking surgery	Surgery incorporated into

		referred to experimental immunotherapy protocol requiring debulking surgery		investigational immunotherapy protocol
G3-3	Metastatic melanoma	Progressive disease despite ipilimumab-nivolumab, liver resection for TIL therapy	Non-anatomic hepatectomy	Surgery integrated into cellular immunotherapy treatment strategy
G3-4	Metastatic RCC	Systemic disease regression under ipilimumab-nivolumab except enlarging adrenal metastasis	Right adrenalectomy	Sanctuary-site progression despite otherwise stable disease
G3-5	Metastatic RCC	Prolonged survival under pembrolizumab complicated by hemorrhagic pancreatic metastasis	Urgent PD	Palliative surgical management during chronic immunotherapy-mediated disease control
G3-6	Metastatic SCLC	Durable response to chemo-immunotherapy complicated by recurrent cholangitis from pancreatic metastasis	Double bypass procedure (hepatico- and gastro-enterostomy)	Surgical management of disease-related complications during prolonged survival

G3-7	Richter transformation/ DLBCL	Multiple prior systemic and cellular therapies with splenic progression under glofitamab	Splenectomy	Surgical management of resistant disease during advanced immunotherapy-based treatment

CCA, cholangiocarcinoma; CRC, colorectal cancer; DLBCL, diffuse large B-cell lymphoma; HCC, hepatocellular carcinoma; HPB, hepato-pancreato-biliary; HPD, hepatopancreatoduodenectomy; ICI, immune checkpoint inhibitor; MSI-H, microsatellite instability-high; PDAC, pancreatic ductal adenocarcinoma; RCC, renal cell carcinoma; SCLC, small-cell lung carcinoma; TIL, tumor-infiltrating lymphocytes.
